# Euthanasia for psychic suffering: the psychopathological (f)law

**DOI:** 10.1192/j.eurpsy.2025.1502

**Published:** 2025-08-26

**Authors:** M. Calmeyn

**Affiliations:** 1 private practice, Loppem Zedelgem; 2 Superior Health Council, Brussels; 3 Postuniversity Center KUL, Kortrijk, Belgium

## Abstract

**Introduction:**

In countries where euthanasia is allowed it seems natural that it can also be applied to persons with psychiatric pathologies, euphemistically called ‘psychic suffering’. Is it that self-evident? In society it still raises ambivalence. In court proceedings it’s almost about psychopathological cases which can’t be solved rightly in a judicial context. These examples are like seismographs detecting special vibrations. Metaphorically speaking several causes are at stake to explain those oscillations. One of them is discussed below. Psychopathology.

**Objectives:**

Psychopathology as such is dynamic – not static. It is a self-organization of complex dynamic systems. The clinical reality and the theoretical grounds corroborate this assumption. This has consequences for the application of euthanasia in case of psychic suffering. Can it still be argued that a psychiatric disease is untreatable? That in the future no change is possible anymore? The objective is to explain that these questions are rhetorical.

**Methods:**

The exploration of two theories leads to the understanding of the proposition, namely that psychopathology is a complex dynamic self-organization.

**Results:**

First, anthropopsychiatry – an integrative theory and praxis of modern psychiatry, philosophy and psychoanalysis with the human(e) as the heart of the matter - states that the human being is intrinsically dynamic and unpredictable. So is the broken man lost in psychopathology. Second, complex living systems are dynamic, with coincidence and uncertainty – in present and future – as core of the systems themselves.

**Conclusions:**

These findings are essential for the theory and practice of euthanasia for psychic suffering. The law only allows it when the psychiatric disease of a person is said to be incurable and not likely to change in the future. From the previous it is clear that this isn’t the case. It’s the flaw of the law in case of euthanasia for psychic suffering.

The importance is to realize these findings and acting accordingly. After all it is a matter of life or death, isn’t it?

**Disclosure of Interest:**

None Declared

